# Chloral

**Published:** 1873-04

**Authors:** 


### CHLORAL

In all its forms benumbs, deadens sensibility, cures nothing, eradicates nothing; it is in reality an opiate; its legitimate use is to gain time and ease pain until eradicative and curative means can be used. The habitual, or even frequent, use of any anodyne will undermine the constitution and shorten life unless antagonizing agencies are employed, or unless favored by some peculiarity of constitution.

